# 肺腺癌的最早期：原位腺癌的病理诊断和临床意义

**DOI:** 10.3779/j.issn.1009-3419.2021.102.35

**Published:** 2021-11-20

**Authors:** 惠康 谢, 岗 陈

**Affiliations:** 1 200433 上海，同济大学附属上海市肺科医院病理科 Department of Pathology, Shanghai Pulmonary Hospital Affiliated to Tongji University, Shanghai 200433, China; 2 200032 上海，复旦大学附属中山医院病理科 Department of Pathology, Zhongshan Hospital Affiliated to Fudan University, Shanghai 200032, China

**Keywords:** 原位腺癌, 世界卫生组织, 分类, Adenocarcinoma *in situ*, World Health Organization, Classification

## Abstract

第五版《世界卫生组织（World Health Organization, WHO）胸部肿瘤分类》（2021版分类）已于2021年5月正式出版，距离上一版2015版WHO分类不过只有短短6年时间。随着近年来低剂量螺旋计算机断层扫描（computed tomography, CT）作为早期肺部肿瘤筛查手段的应用，肺腺癌早已成为许多医院手术治疗的优势病种。WHO分类作为指导病理诊断的权威指南，任何一处细微的变动都牵扯病理、临床及患者的心弦。原位腺癌为2015版分类新增加的腺癌诊断，也是近年来国内外临床及研究的焦点，因新版分类对其目录位置进行了一定调整，由此在临床医师及患者中产生了“原位腺癌被剔除出恶性肿瘤范畴”的巨大争议和讨论，本文将就肺原位腺癌诊断的来龙去脉，新分类目录调整以及原位腺癌良恶性的问题进行简要说明。

## Abstract (en)

The International Agency for Research on Cancer (IARC) published the World Health Organization (WHO) classification of thoracic tumors (5^th^ edition) in May 2021, only six years after the 4^th^ edition of WHO Classification. With the application of low-dose spiral computed tomography (CT) as an early screening method for lung tumors in recent years, lung adenocarcinoma has become the main type of disease in many hospital surgical treatments. The WHO classification serves as the authoritative guide for pathological diagnosis, and any slight change in the classification is at the heart of pathologists, clinicians and patients. Adenocarcinoma *in situ* is a newly added type of adenocarcinoma diagnosis in the 4^th^ edition of the WHO classification, and it is also the focus of clinical treatment and research at home and abroad in recent years. Because its catalog position has been adjusted in the 5^th^ edition of the WHO classification, there has been a huge controversy and discussion among clinicians and patients that "adenocarcinoma *in situ* was excluded from the category of malignant tumors". This article will briefly explain the origin of the diagnosis of lung adenocarcinoma *in situ*, the adjustment of the new classification catalog, and whether adenocarcinoma *in situ* is benign or malignant.

## 1 原位腺癌

病理诊断的目的是为了配合临床治疗，随着精准化治疗的推进，肺腺癌的分类也越来越精细化。1967年分类仅将肺腺癌分为支气管源性和肺泡源性；1981年分类将肺腺癌亚型进一步拓展为腺泡型、乳头型、细支气管肺泡癌（bronchioloalveolar carcinoma, BAC）、实体性腺癌四型。随后的Noguchi分类^[1-3]^通过不同亚型的混合，对腺癌的不同阶段进行分类，虽然该分类未被世界卫生组织（World Health Organization, WHO）分类直接采用，但该分类与临床预后直接相关，所以该分类理念对后续的WHO分类影响深远。2011年国际肺癌研究协会/美国胸科学会/欧洲呼吸学会（International Association for the Study of Lung Cancer/American Thoracic Society/European Respiratory Society, IASLC/ATS/ERS）多学科分类^[4]^对肺腺癌分类进行了大刀阔斧的改革，多学科改革的重点之一便是去除了细支气管肺泡癌的诊断，新增了原位腺癌、微浸润腺癌、微乳头腺癌的诊断。原位腺癌源自2004版分类^[5]^细支气管肺泡癌的诊断，2004版对于BAC的定义为“显示肿瘤细胞沿着尚存的肺泡架构生长，无间质、血管、胸膜侵袭的证据”。从中我们可以明显感受到后续原位腺癌诊断定义的影子，之所以对原诊断进行更改，主要原因在于实际病理诊断应用层面BAC的诊断过于混乱，其诊断跨度包含了多学科分类中的原位腺癌、微浸润腺癌、贴壁型为主腺癌及浸润性粘液腺癌这些不同浸润阶段及预后的腺癌类型。2015版分类完整吸收了2011版多学科分类中的框架，其中原位腺癌被定义为“腺癌细胞完全沿肺泡壁生长（贴壁样），未出现间质、血管或胸膜侵犯，未混合有腺泡、乳头、实性或微乳头成分，肺泡腔内无肿瘤细胞，肿瘤最大直径≤3 cm”。通过对于原位腺癌更为严格的诊断定义和要求，2015版分类将原位腺癌框定在了一个预后层面绝对安全的诊断范围内，使得临床能够以更为统一的治疗手段对待这一亚类的早期肺腺癌。其临床意义在于通过CT影像直观地判断肿瘤状态，决定治疗手段，2021版新分类则完全继承了2015版对于原位腺癌的定义，形态学诊断标准上亦未发生任何改变^[1, 2]^。

## 2 新版WHO目录调整

2021版分类在目录编排上进行了较大的调整，其中一大改革是将所有同一组织来源的肿瘤从良性——不典型增生——原位癌——浸润癌进行了重新的排列整理，这样的好处是让读者可以仅通过目录的阅读便可直观感受到不同肿瘤间的良恶性程度排序。有读者发现在2021版分类内，WHO将原位腺癌由原2015版分类腺癌（adenocarcinomas）子目录下，移至了前驱腺性病变（precursor glandular lesions）子目录下，与不典型腺瘤样增生放在了并列的位置，而微浸润腺癌仍然在腺癌子目录下，由此得出了原位腺癌不再是癌这样一种“结论”，这种理解是片面的，WHO分类是一个临床病理分类，而不是单一的组织学分类，是从便于临床治疗的生物学行为的角度进行了编排，原位腺癌的性质并没有改变。通过对于新版分类更为彻底广泛的翻阅，我们可以明确地发现这种目录上的编排更新并非针对某单一癌种，而是针对2021版分类中的全部肿瘤类型，如：旧版WHO肺肿瘤分类中的肺腺癌、肺鳞状细胞癌、肺神经内分泌肿瘤目录下的非典型腺瘤样增生/原位腺癌、鳞状上皮不典型增生/原位鳞状细胞癌、弥漫性特发性肺神经内分泌细胞增生，新版WHO分类中均调整至了对应肿瘤目录中的前驱病变处。与原位腺癌诊断地位相同的原位鳞状细胞癌更是经历了从2004版分类“前驱病变”，到2015版分类移至“浸润前病变”，于2021版分类回归“前驱病变”的数次目录位置调整。所以不应仅以目录位置的调整对肿瘤的良恶性作出不恰当的判断。

## 3 ICD-O编码

ICD（International Classification of Diseases）为WHO主导开发的诊断各类疾病的统计术语集合，被全球医疗、科研、政府以及保险机构广泛使用。ICD-O则为专门针对肿瘤性病变开发的编码系统，该编码包含两个部分：前半部分为病变部位编码：T编码（topography），后半部分为形态学特征编码：M编码（morphology）。顾名思义，T编码通过69类组织器官和329个具体部位，可以精确定位肿瘤发病的具体解剖位置，M编码则进一步分为“/”前四位的细胞学类型代码和“/”后一位的肿瘤良恶性编码。WHO分类中标注的ICD-O编码为后半部分的M编码，其中决定肿瘤良恶性程度的则是“/”后的肿瘤良恶性编码。该编码具体意义见表 1。通过对比，我们发现，2004版分类中BAC的肿瘤良恶性编码为3（原发性恶性肿瘤），2015版分类中无论是粘液型还是非粘液型原位腺癌，其良恶性编码均更改为2（原位癌），而2021版分类原位腺癌的肿瘤良恶性编码对2015版进行了完全的继承，均标记为2。所以通过几版分类ICD-O的对比我们可以发现，2015版和2021版分类对于原位腺癌是否为“癌”这一概念，有着清晰的回答，即原位腺癌是癌，但是处于原位状态，两版分类没有任何差别。

**表 1:** ICD-O编码内肿瘤良恶性编码 Tumor benign and malignant codes within ICD-O codes

0	良性
1	良恶性无法区分
2	原位癌
3	原发性恶性肿瘤
6	转移性恶性肿瘤
9	恶性肿瘤无法区分转移与否
ICD: International Classification of Diseases.	

综上，原位腺癌为2015版WHO新出现的诊断，因2021版分类整体目录调整其目录位置发展了一定的改变，通过ICD-O编码中的良恶性编码可以明确原位腺癌的肿瘤良恶性认定未发生任何改变，仍然为癌，且处于原位状态。
